# DbKB a knowledge graph dataset for diabetes: A system biology approach

**DOI:** 10.1016/j.dib.2023.110003

**Published:** 2024-01-03

**Authors:** Rauf Ahmed Shams Malick, Siraj Munir, Syed Imran Jami, Shoaib Rauf, Stefano Ferretti, Hocine Cherifi

**Affiliations:** a Department of Computer Science, National University of Computer and Emerging Sciences, Karachi, Pakistan; b Department of Applied and Pure Sciences, University of Urbino Carlo Bo, Urbino, Italy; c Department of Computer Science, Mohammad Ali Jinnah University, Karachi, Pakistan; d ICB UMR 6303 CNRS, University of Burgundy, Dijon, France

**Keywords:** Information representation, Semantics, Querying, Diabetic knowledge representation

## Abstract

Diabetes has emerged as a prevalent disease, affecting millions of individuals annually according to statistics. Numerous studies have delved into identifying key genes implicated in the causal mechanisms of diabetes. This paper specifically concentrates on 20 functional genes identified in various studies contributing to the complexities associated with Type 2 diabetes (T2D), encompassing complications such as nephropathy, retinopathy, cardiovascular disorders, and foot ulcers. These functional genes serve as a foundation for identifying regulatory genes, their regulators, and protein-protein interactions.

The current study introduces a multi-layer Knowledge Graph (DbKB based on MSNMD: Multi-Scale Network Model for Diabetes), encompassing biological networks such as gene regulatory networks and protein-protein interaction networks. This Knowledge Graph facilitates the visualization and querying of inherent relationships between biological networks associated with diabetes, enabling the retrieval of regulatory genes, functional genes, interacting proteins, and their relationships.

Through the integration of biologically relevant genetic, molecular, and regulatory information, we can scrutinize interactions among T2D candidate genes [1] and ascertain diseased genes [2]. The first layer of regulators comprises direct regulators to the functional genes, sourced from the TRRUST database in the human transcription factors dataset, thereby forming a multi-layered directed graph. A comprehensive exploration of these direct regulators reveals a total of 875 regulatory transcription factors, constituting the initial layer of regulating transcription factors. Moving to the second layer, we identify 550 regulatory genes.

These functional genes engage with other proteins to form complexes, exhibiting specific functions. Leveraging these layers, we construct a Knowledge Graph aimed at identifying interaction-driven sub-networks involving (i) regulating functional genes, (ii) functional genes, and (iii) protein-protein interactions.

Specification Table

**Table:** 

Subject	Health and Medical Science, Diabetes, Artificial Intelligence
Specific subject area	A Knowledge Graph-based Dataset for Type 2 Diabetes Patients
Data format	Graph Representation and Filtered
Type of data	Knowledge Graph, Dump file
Data collection	The dataset was extracted from the Gene Regulatory Network (TRUSST DB) and Protein-Protein Interaction Network (String DB) then we filtered data based on T2D patients and identified 20 Genes. The details of data extraction and processing are as follows. • For data extraction, we used Python programming language-based data scraper and retrieved 875 and 550 regulatory Transcription Factor (TF) and regulatory genes respectively. • Further, to make this data more useful for domain experts and researchers we model Knowledge Graph. The Knowledge Graph holds the semantic representation using a graph model. • For modeling Knowledge Graph, we used Neo4j one of the greatest tool for graph databases.^1^
Data source location	• Institution: Department of Pure and Applied Sciences, University of Urbino and Department of Computer Science, National University of Computer and Emerging Sciences. • City/Province/Region: Urbino, Pesaro and Urbino, Marche, Dijon, and Karachi, Sindh • Country: Italy, France, and Pakistan
Data accessibility	Direct URL to data: https://zenodo.org/records/10402281 Instructions for accessing these data: The dataset repository contains a dump file that can directly be imported to Neo4j Desktop available at https://neo4j.com/download/. Once you have downloaded and installed Neo4j simply follow the instructions at https://neo4j.com/docs/desktop-manual/current/operations/create-from-dump/#:~:text=Once%20you%20have%20a%20dump,when%20creating%20a%20new%20DBMS.

1 https://neo4j.com/.

## 1 Value of the Data


 

• The Knowledge Graph facilitates users in seamlessly constructing an information and knowledge structure based on retrieved data from various databases, including TRUSST, STRING, GO, and GEO. The TRUSST database holds regulatory information, while the STRING database contains details about functional proteins. DbKB integrates interactions among gene-protein and protein-protein within its framework.
• Causality analysis studies stand to gain significant insights from the comprehensive dataset provided in DbKB. The platform is designed as a multiscale information structure, enabling users to correlate biological functions with groups of proteins alongside regulatory details. For studies focused on identifying key genes in specific pathways, DbKB proves beneficial by assessing centrality and associations with disease orientation.
• DbKB empowers users to construct knowledge structures through the enrichment of interactions and nodes, encompassing genes and proteins. This approach allows for complex multidimensional analysis, opening novel ventures for introducing new types of interactions for future investigations.
• Another interesting contribution of DbKB is drug design. Studies will harness the structured and integrated data offered by DbKB, providing a valuable resource for advancing research in this field.

## 2 Background

Diabetes has emerged as a severe and complex disease affecting a substantial portion of the population in recent decades. This chronic condition stems from elevated blood glucose levels and the altered metabolism of fats and proteins [3]. Type 2 diabetes, similarly, is influenced by genetic factors, environmental agents, and their intricate interactions [6].

To comprehensively study and comprehend the key factors contributing to diabetes, it is imperative to model the available information in a manner that facilitates the identification of its causes and underlying facts. DbKB introduces a model that interconnects all of them via entities, concepts, and their relationships within a Knowledge Graph. This model serves as a framework for data integration, offering insights into the data. Graphs have proven to be an interesting tool for consolidating knowledge and data globally.

Several studies have highlighted various genes as 'crucial' for diabetes [4,5], with distinct genes contributing to different complications. Seven genes have been identified as significant for all five diabetic complications: nephropathy, neuropathy, retinopathy, cardiovascular problems, and atherosclerosis [6]. Understanding the development of cardiovascular complexity alongside diabetes involves the significance of 172 genes [6]. Recognizing the need for an integrated repository of diabetic data centered on key genes, numerous research papers and studies have been published in the past two decades [18,19]. These papers provide empirical evidence for identifying key genes and validate this through gene expression analysis in patients versus controls.

The availability of diverse heterogeneous biological data has enabled the study of diseases from a multiscale perspective [7], [8], [9]. While the utilization of biological networks to study diabetes and its complexities is expected to grow, each study necessitates a sequence of data retrieval pipelines from multiple databases to establish the desired network. Moreover, in the case of multi or multiplex networks, the data retrieval pipeline becomes more intricate and laborious. This emphasizes the need to develop a common database capable of offering desired data from multiple perspectives within a multi-network study framework.

## 3 Data Description

**Fig. 1:**
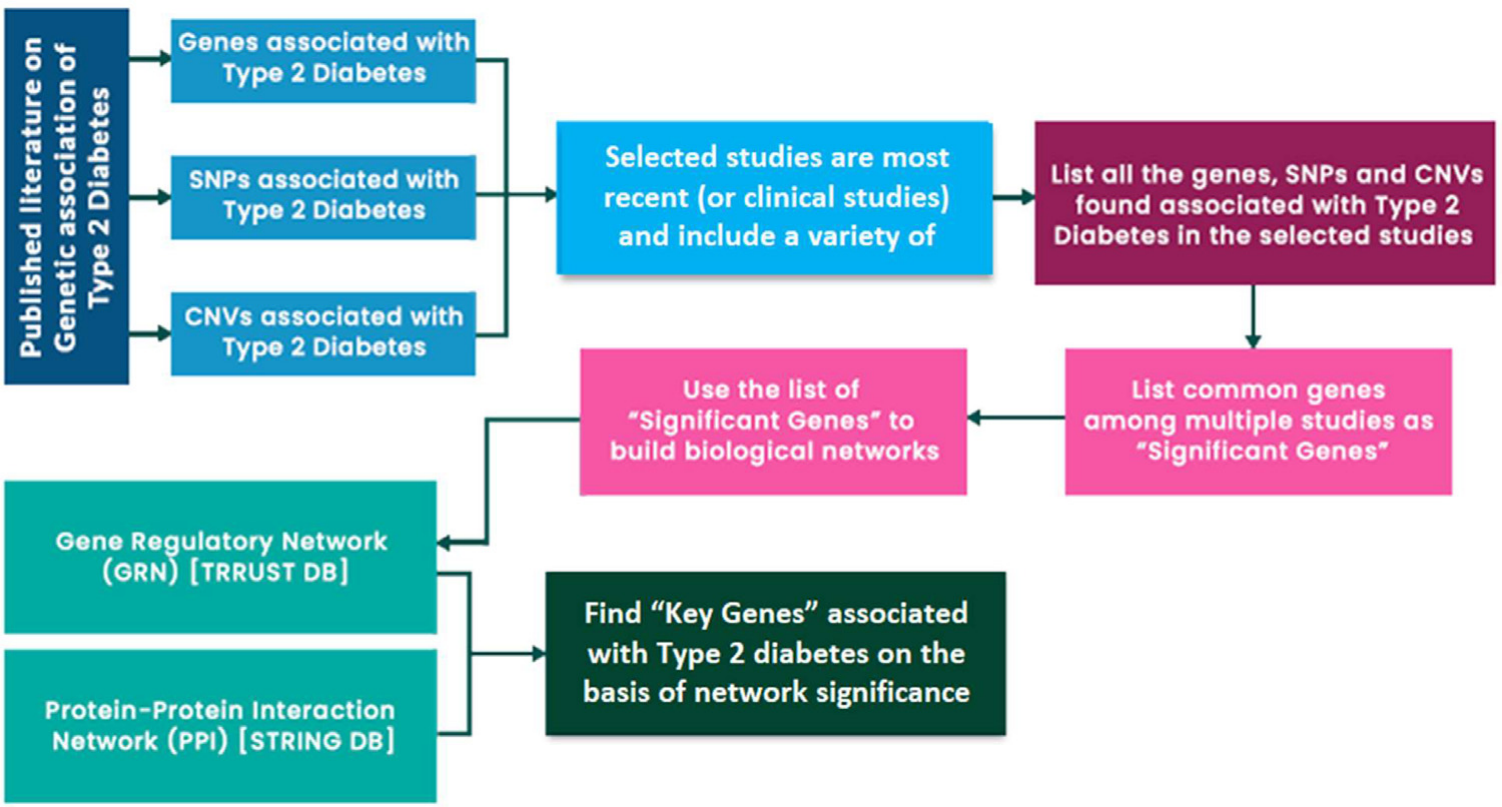
For extracting regulatory genes and proteins, we devised a data acquisition pipeline to systematically gather relevant information from TRRUST, STRING, and GEO databases. To streamline the process and focus on genes of particular significance.

We leverage biological networks to explore diabetes and its associated complexities from a multiscale perspective. Graph-based databases offer effective means to represent the intricate interactions and relationships within biological networks. Neo4j serves as a platform for maintaining graph databases and providing tools for network analysis, data retrieval, and visualization. In our database, we focused exclusively on genes that have been consistently reported as significant across multiple studies and populations. To identify key functional genes, we employed multiple filters during the data acquisition as outlined in Fig. 1. Based on our criteria, we identified 20 genes labeled as ‘key’ functional genes.

However, for modeling of Knowledge Graph, we extrapolated three databases, namely TRRUST, STRING, and GEO. The TRRUST database provides information about 800 regulatory genes TFs (Transcription Factors) and their 8444 target genes which are being regulated by these TFs in humans. This data can be used to identify human regulatory genes [10]. Sentence-based text mining followed by manual curation is used to develop the TRRUST database [11]. Regulatory genes (Transcription Factors TFs) were retrieved from TRRUST DB using Python scripting.

The STRING database has 24,584,628 numbers of proteins from 5090 organisms. This database contains protein-protein interactions [12], which provides information about direct (physical) interaction and indirect (functional) interaction between proteins, which helps to understand the complex network of cellular interactions taking place within the cell [13]. Protein-protein interactions of “functional genes” were retrieved using the STRING API through Python scripting.

GEO database provides gene expression levels of cells [14]. It is the largest database of gene expression data which is publicly available and accessible. It currently holds over 30,000 gene expression datasets for over 100 organisms [15]. The GEO dataset was used to analyze the gene expression profiles of diabetic vs. control subjects. Table 1 shows the inherited information about the Knowledge Graph. The presented Knowledge Graph is composed of 6918 nodes having 5 different genres of 4855 interactions.

**Table 1:** Summary of knowledge graph.

Parameters	Values
Relationships count and types	4855 (consisting of 5 types: Has_Protien_Interaction, Has_Referecne_No, Has_Regulator, Has_Target_Protien, Is_At_Layer)
Label	5 (Types of nodes)
Total nodes	6918

## 4 Experimental Design, Materials, and Methods

The experimental design and method are divided into five protocols.

### 4.1 Basic protocol 1: integration of biological sources for the development of a multi-scale network model of diabetes (MSNMD)

**Fig. 2:**
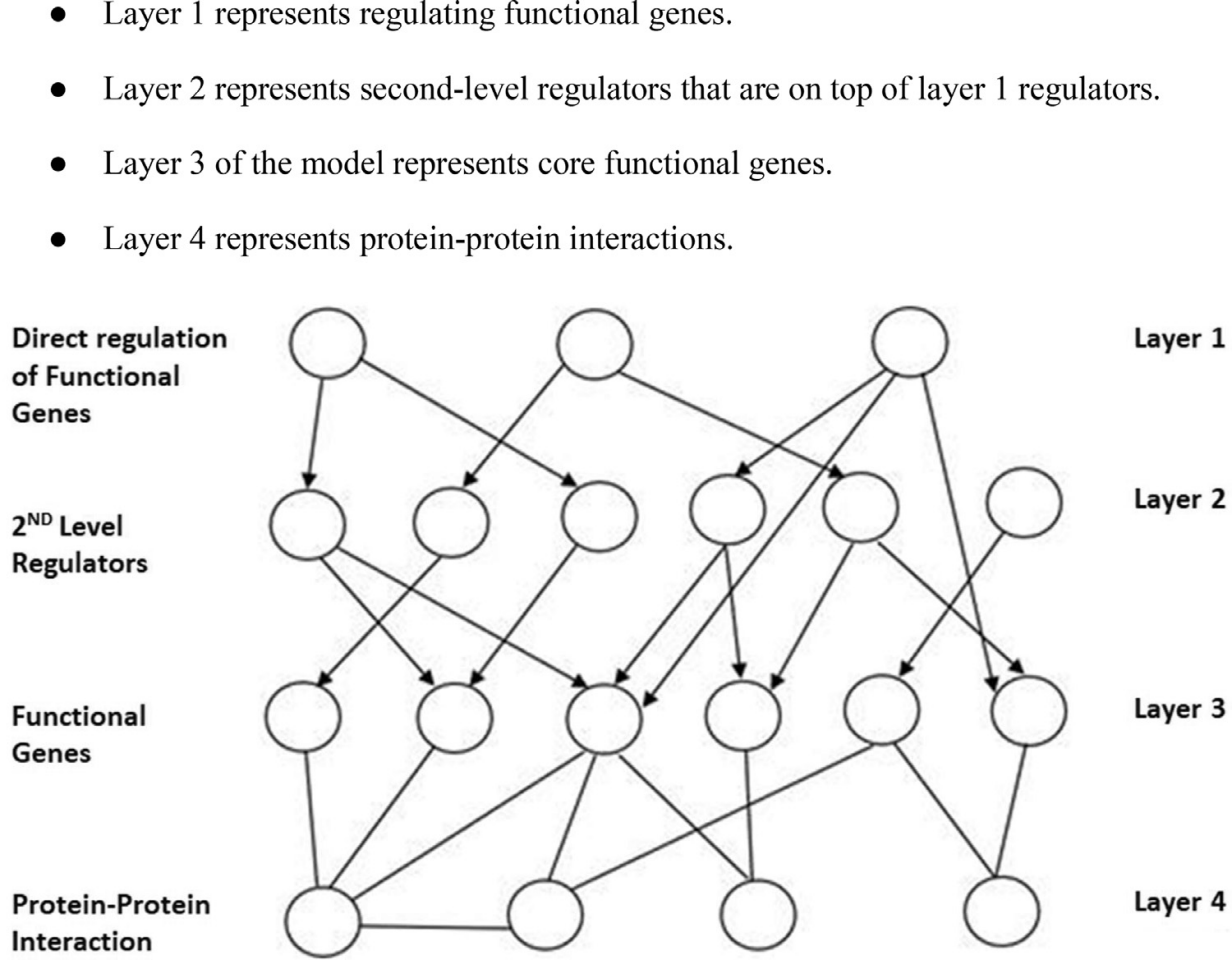
Multi-Scale Model of Diabetes: Predicts the metabolic and inflammatory processes on the commencement and progression of T2D by studying the systematic interactions of the biological mechanisms.

To study diabetes, particularly Type 2 diabetes (T2D), a system biology approach is used that can help examine the complex nature of this disease through a network [1]. A candidate molecular network can be generated through knowledge and statistics-based systematic analysis of high throughput molecular data of normal and diseased patients [1]. This multi-scale network model is developed, which consists of the Gene Regulatory Network (GRN) layer, Functional Genes layer, and Protein-Protein Interaction (PPI) layer. By integrating biologically related genetic, molecular, and regulatory information, we can evaluate the interactions between the T2D candidate genes [1] and determine diseased genes [2]. Fig. 2 shows the multi-scale network model that is reflected in the database through the Neo4j tool.

• Layer 1 represents regulating functional genes.
• Layer 2 represents second-level regulators that are on top of layer 1 regulators.
• Layer 3 of the model represents core functional genes.
• Layer 4 represents protein-protein interactions.

The direct regulators to the functional genes, found in the TRRUST database in the human transcription factors dataset, formed the first layer of regulators hence a directed graph. Furthermore, these direct regulators are investigated, and a total of 875 regulatory transcription factors are found, regulating the first layer of regulators, and 550 regulatory genes are found in the second layer of regulators.

### 4.2 Basic protocol 2: development or identification of functional genes

Numerous functional relations exist between genes in a cell process [16]. These are genes that were identified as directly contributing to multiple complexities. These complexities of diabetes and found by experimental evidence. Hence a functional unit of genes is clustered to analyze arrangements that may help to visualize all three levels [16]. The genes of a functional group tend to co-localize within the given space [16]. By examining their genomes, these genes can be detected and clarified by further evidence.

### 4.3 Basic protocol 3: identification of gene regulatory network

Gene Regulatory Networks (GRN) comprise the interaction of regulatory genes within target genes and the mode of regulation is represented as activators or repressors. Therefore, it can be referred to as a network of regulators. Regulatory genes have a directed role in regulating the target gene; consequently, they can be represented through directed graphs.

The regulatory gene regulations can be predicted using a systematic approach which can be used to identify the interactions between the components. This information can further be used to model GRNs [17].

### 4.4 Basic protocol 4: identification protein-protein interaction (PPI)

Protein-protein interaction (PPI) is essential in almost every cell function and plays a crucial role in predicting the function of a protein and the drug ability of molecules. Proteins collaborate with other proteins in a GRN to form complexes, participate in functional processes within a cell cycle and regulate the target gene expression [17]. In connection with GRN, PPI is used as an undirected graph as shown in Fig. 2.

### 4.5 Basic protocol 5: development of knowledge graph (MSNMD)

**Fig. 3:**
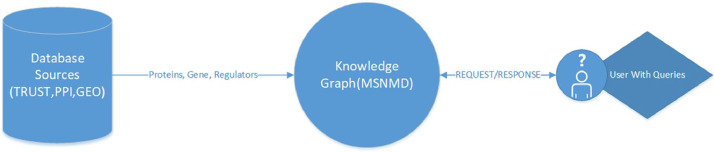
Pipeline for the construction of Knowledge Graph (MSNMD). Data has been collected from different renowned databases and combined for ease of better representation and understandability.

Datasets are generated as a result of several procedures. To make these complex datasets accessible for further use, graphs are considered for natural representation. Neo4j is a top-notch graph database used for several use cases, including modeling biological datasets, Knowledge Graphs, ontologies, etc. Fig. 3 depicts a pipeline for constructing a Knowledge Graph (MSNMD) from different biological databases so that it can be queried.

**Fig. 4:**
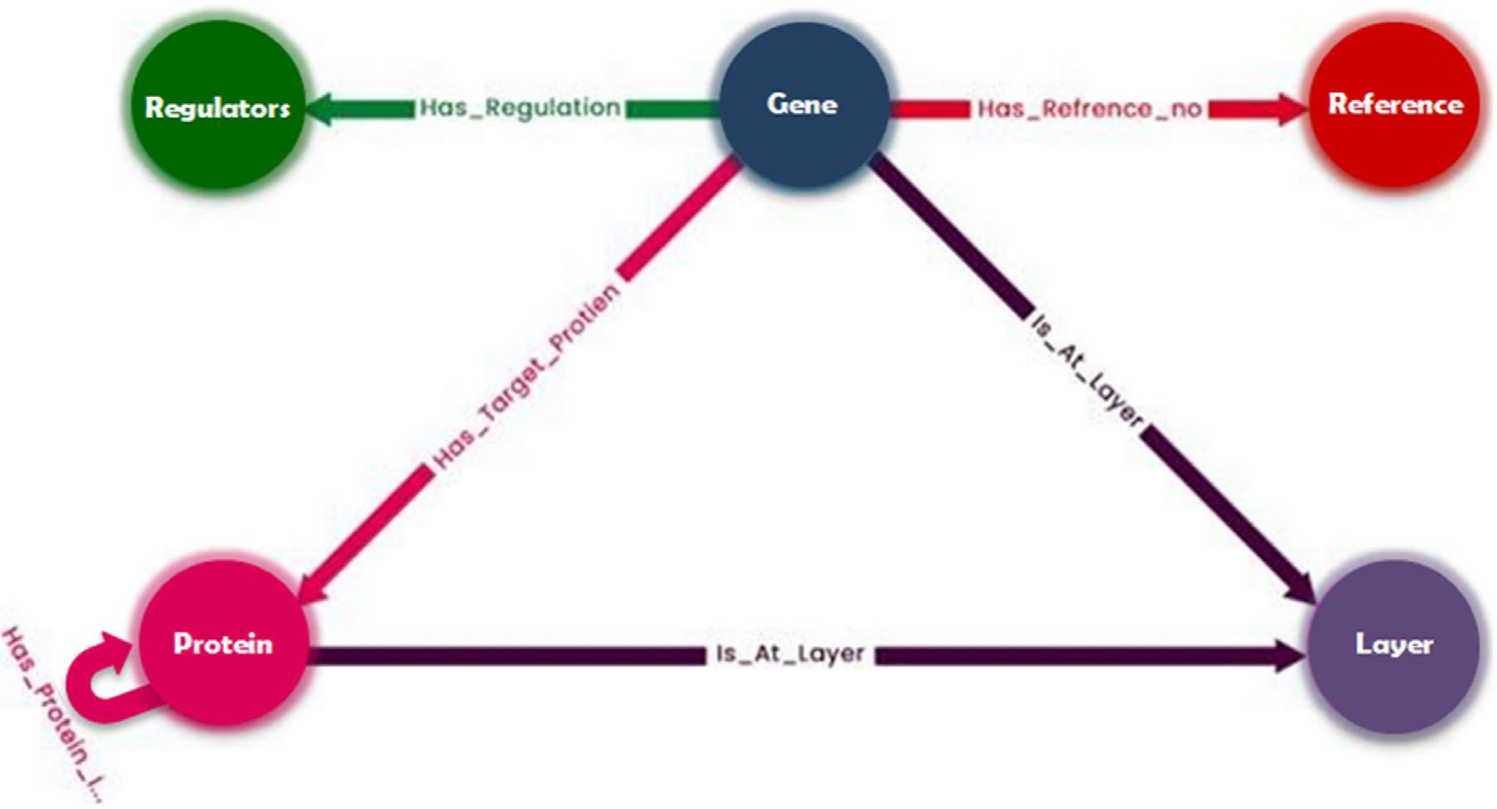
Knowledge Graph Data Model: Each gene has a link or relationship with other nodes in the Knowledge Graph. Each gene has a target protein, layer, regulator, and reference number.

Fig. 4 shows the graph data model, where each color represents a node and relationship type. A gene has a reference number by which it can be referenced. A particular gene is regulated by regulators. It also has a target protein and is present at a certain layer. Proteins are present in separate layers. This layer shows the interaction between different proteins.

**Fig. 5:**
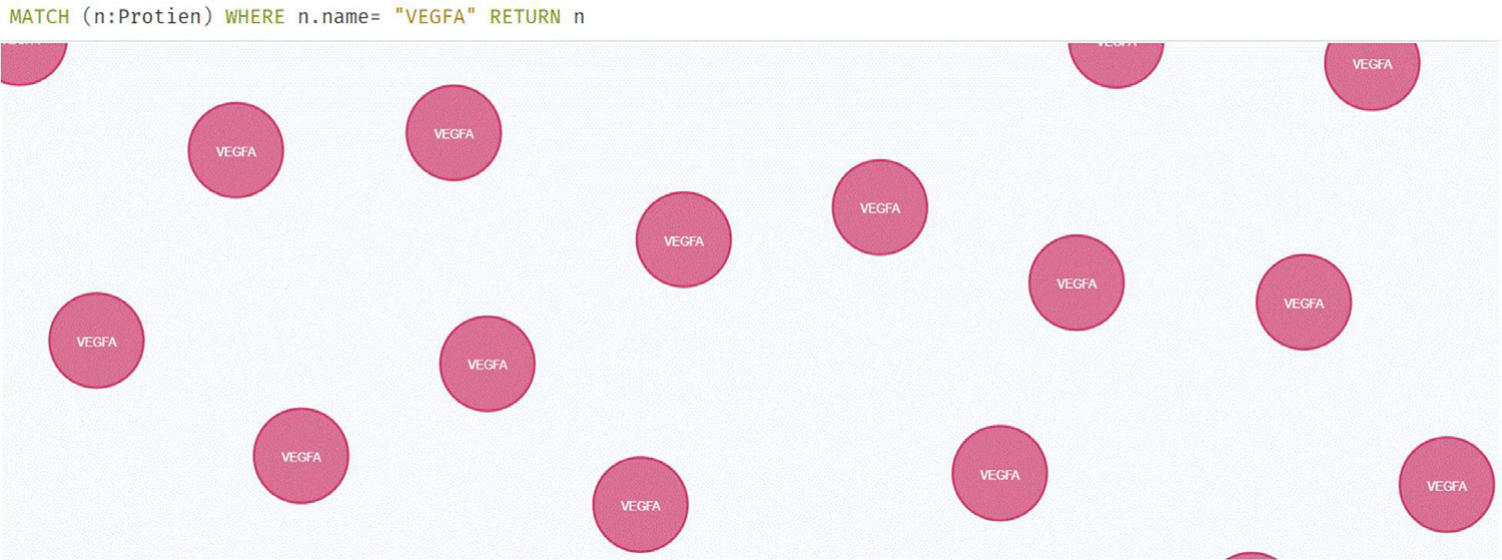
Protein filtering based on type. Proposed representation can also aid in the extraction of curated data. Here for the sample, we have filtered data based on the protein VEGFA.

**Fig. 6:**
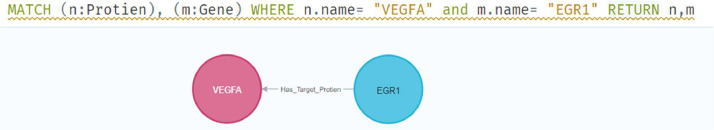
Protein-gene interaction has been dealt with their inherited relationship. However, this information is also filterable i.e. we can check each protein and gene interaction individually at each level (if exists).

**Fig. 7:**
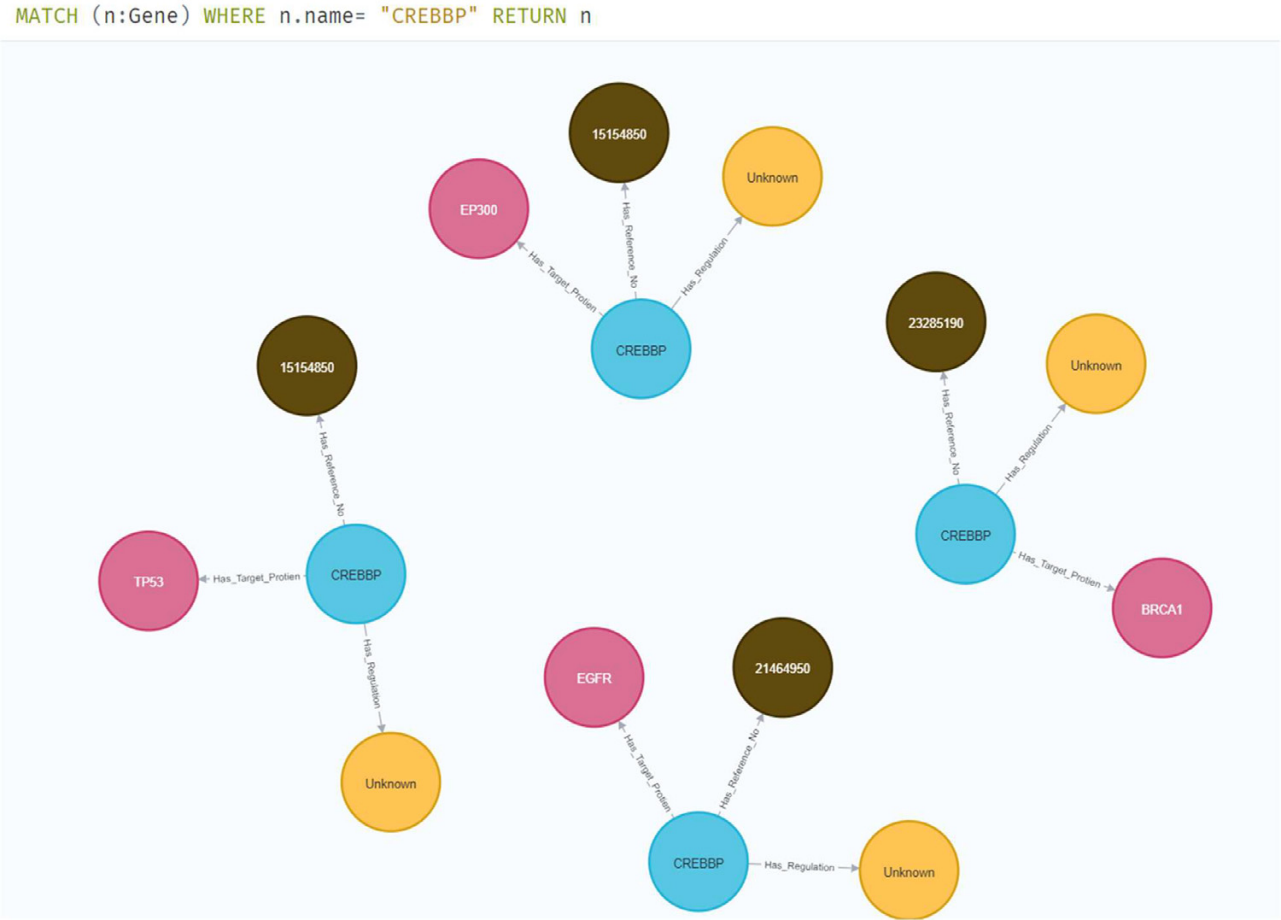
Specific gene interaction with different proteins and activation type along reference.

The objective of Knowledge Graph-based representation is twofold. Graph databases tend to represent inherent relationships among the biological networks, and they also help a user perform interactive queries. Queries are not limited to separate network layers. They can be used to retrieve information from multiple layers at once. This includes PPI+Functional Layer, GRN+Functional Layer, GRN+Functional Layer+PPI. Various combinations can be applied to retrieve relevant data that was previously difficult to model, manipulate, retrieve, and visualize. The multiscale model of the Knowledge Graph makes a user independent of retrieving data from one layer at a time. Here Fig. 5, Fig. 6, Fig. 7 depict the queryable information along with queries.

## Limitations

The knowledge graph DbKB needs enrichment and updates after some time as new datasets are available and enrichment is a continuous process. Complex information has to be integrated further including pathway information from KEGG, protein structural information from PDB, and cancer information from SNP and Onco-related databases.

Diabetes is a common disease that has been rapidly growing over the years. Analyzing and understanding its pandemic is crucial for research and the medical community. Hence in this paper, we presented MSNMD with a Knowledge Graph that is the first of its kind. By integrating a multi-source pipeline, we were able to present a holistic view of T2D using useful protocols. Moreover, it represents the multiscale networks contributing to T2D. However, as suggested above we require more data sources to be integrated so that we can have a universal view of pandemic.

## Ethics Statement

The current work does not involve human subjects, animal experiments, or any data collected from social media platforms.

## CRediT authorship contribution statement

**Rauf Ahmed Shams Malick:** Conceptualization, Methodology, Writing – original draft, Investigation. **Siraj Munir:** Methodology, Validation, Writing – review & editing. **Syed Imran Jami:** Writing – review & editing. **Shoaib Rauf:** . **Stefano Ferretti:** Writing – review & editing. **Hocine Cherifi:** Writing – review & editing.

## Data Availability

Gene-ProtienDataset (Original data) (Zenodo)
